# Efficacy of a Smoking Cessation Program for Underserved Ethnic Minority Communities: Results of a Smoking Cessation Trial

**DOI:** 10.3389/ijph.2023.1605739

**Published:** 2023-06-20

**Authors:** Payam Sheikhattari, Jummai Apata, Lisa Bleich, Farin Kamangar, Shervin Assari

**Affiliations:** ^1^ Department of Behavioral Health Science, School of Community Health and Policy, Morgan State University, Baltimore, MD, United States; ^2^ Prevention Sciences Research Center, Morgan State University, Baltimore, MD, United States; ^3^ Signal Fire Consulting, Baltimore, MD, United States; ^4^ Office of Research Administration, Morgan State University, Baltimore, MD, United States; ^5^ Department of Family Medicine, Charles R. Drew University of Medicine and Science, Los Angeles, CA, United States; ^6^ Department of Urban Public Health, Charles R. Drew University of Medicine and Science, Los Angeles, CA, United States

**Keywords:** smoking cessation, health disparities, peer-motivation, participatory research, underserved ethnic minorities

## Abstract

**Objectives:** Using a participatory research approach, this study reports the efficacy of the Communities Engaged and Advocating for a Smoke-free Environment (CEASE)-4 intervention offered by the local peers.

**Methods:** CEASE-4 is a theory-based tobacco-cessation intervention, tailored to the needs of underserved populations. 842 tobacco users self-selected into: a) self-help (*n* = 472), b) single-session class (*n* = 163), and c) four-session class (*n* = 207). While self-help group only received educational materials, curriculum for other arms was built on the social cognitive, motivational interviewing, and trans-theoretical- frameworks. Participants could also receive nicotine replacement therapy (NRT). Outcome was self-reported smoking cessation measured 12 weeks after completion of the intervention, validated by exhaled carbon monoxide (CO) test.

**Results:** Quit rate was statistically different across groups, with highest quit rate in four-session and lowest quit rate in self-help arm. Cessation rates at follow up (12 weeks after completion of the intervention) were 2.3% in the self-help arm, 6.1% in the single-session arm and 13.0% in the four-session arm.

**Conclusion:** While theory-based smoking cessation services are effective for underserved populations, four-session curriculum might be superior to a single session program.

## Introduction

Tobacco use is a major public health problem in the United States and globally. With about half a million tobacco-related deaths every year, tobacco is the foremost preventable cause of death in the U.S [1]. According to the Centers for Disease Control (CDC), nearly 12 of every 100 U.S. adults used tobacco in 2021 [1]. Since the first Surgeon General’s Report published in 1964, tobacco smoking has been identified as a leading cause of lung cancer and overall mortality in the US [2]. The health impact of tobacco use is, however, not limited to lung cancer as it affects almost all body organs [2]. Tobacco smoking also exerts a high economic burden on communities and families. Tobacco smoking imposes more than $300 billion in direct (healthcare) and indirect (productivity loss) costs to the U.S. government each year [3]. Direct healthcare costs are 40% higher for smokers than nonsmokers [4]. Thus, there is a need for development of effective treatment programs that promote tobacco cessation across diverse U.S. communities.

Through various strategies and policies, the U.S. has experienced a decline in overall tobacco use. For example, cigarette smoking prevalence declined from 42.4% in 1965 to 11.5% in 2021 [1, 2]. However, the decline in tobacco use has been unequal across economic and ethnic sub-populations, resulting in a larger prevalence of tobacco use in socially disadvantaged groups defined by race, ethnicity, and socioeconomic status (SES) in more recent years [2]. As shown by epidemiological studies that analyzed secular trends of tobacco use over time by race, ethnicity, and SES, while smoking showed a steep decline in high SES and non-Latino White populations, the poor, underserved, and racial/ethnic minorities in contrast remained at risk of high levels of tobacco smoking [3, 5, 6]. This is notable, given the poor access to treatment services of vulnerable populations (as measured by race, ethnicity, and SES), increasing the propensity for developing undesired consequences of tobacco use which remain disproportionately high in underserved communities [7]. Thus, there is a need to develop tailored interventions that show high efficacy and acceptability and can be implemented in underserved communities.

Communities Engaged and Advocating for a Smoke-Free Environment (CEASE) is a community driven smoking cessation initiative that evolved through collaboration between Morgan State University and residents of the Southwest Baltimore communities with a mission to educate, encourage, and support individuals to choose a smoke-free lifestyle. CEASE is a theory based culturally appropriate behavioral modification intervention that is centered around peer-led programs as an effective strategy for smoking cessation among underserved populations. CEASE is designed based on health behavior models [8, 9] such as Social Cognitive Theory [10–12] and trans-theoretical (TTM) framework [13, 14]. CEASE, initiated in 2007, was specifically designed and implemented for predominantly Black communities with lower household income. In CEASE, peer-motivators, mostly ex-smokers, are trained to facilitate individual and group counselling sessions. CEASE was implemented as a Community Based Participatory Research (CBPR) project in response to the needs assessment involving community stakeholders [15]. CEASE has multiple phases, and the current study is on CEASE-4. CEASE-1 [8, 9, 15], CEASE-2 [8, 9, 16] and CEASE-3 [8, 9, 16] have shown efficacy of this program in different settings, using different modes of intervention. The CEASE 1 to 3 all used a 12-week curricular intervention, while the CEASE-4 is an attempt to have a fewer number of sessions which may be essential for cost reduction, sustainability, and scale up of the program.

We aimed to a) test the effectiveness of the CEASE-4 program which utilized a one-session and a four-session curriculum, in a racial/ethnic minority-majority community with low socioeconomic status and b) explore correlates of response to the intervention. Similar to past versions of CEASE [8, 9, 16], we expected an increase in quitting as a result of CEASE-4, nevertheless, given that CEASE -4 used fewer sessions, we anticipated mild- to moderate effects.

## Methods

### Study Design

We used a quasi-experimental design to compare the efficacy of a single-session and a four-session smoking cessation intervention, which were both developed from the previous 12-session iteration of the CEASE intervention, with a self-help intervention as a control. The study was conducted between 2015 and 2017.

### Study Population and Sample Size

The setting of this study was low-income minority-majority communities in Baltimore City. Participants from these communities were adult smokers who consented to participate in the program. A total sample of 842 participants self-selected into any of the study’s three arms.

### Participant Recruitment

Participants who were 18 years and older and were current smokers were recruited for the study. Current smoking status was defined as smoking at least three cigarettes per day in the past week.

Participants were recruited using two main approaches: from a community survey and through a targeted recruitment approach. Peer motivators attended certain community events (i.e., farmers’ market, church services, health fairs, etc.) where they approached and asked individuals at the event to take a brief web-based community survey on tobacco, using iPads. Survey respondents who were adults and reported regularly smoking at least three cigarettes a day, were invited to participate in one of three study arms. In the targeted recruitment, peer motivators visited locations in the communities generally known for having smokers outside, such as bars, restaurants, street corners, and clinics. They approached people smoking outside these locations and invited them to participate in one of the three arms of the study if they were eligible. Interested participants completed the community survey and were scheduled for the classes. Additionally, recruited participants and program partners could refer other eligible smokers to the program who could contact peer motivators to complete the survey and join a class if eligible. All community survey respondents were offered carbon monoxide (CO) breath test at the time of completing the survey.

### Intervention Arms

Participants self-selected into the following three study arms: a four-session group counseling (*n* = 207); a single-session group counseling (*n* = 163); or a self-help group that received educational materials (control group: *n* = 472). The smoking cessation sessions were conducted in community venues and were facilitated by experienced peer motivators who had worked in previous CEASE interventions. As in earlier phases, all peer motivators had at least a high school diploma, were former smokers, and had abstained from smoking for at least 1 year.

The curriculum had been enhanced over the course of the previous three phases. It included motivation enhancement, preparation for quitting, tobacco cessation, use of Nicotine Replacement Therapy (NRT), and relapse prevention. In the single-session arm, participants attended a class lasting 1.5 h, based on a very compressed version of the 12-session approach and facilitated by an experienced peer motivator. Participants in the four-session arm attended weekly sessions with an appropriately compressed curriculum. Both the single-session and four-session participants received a “quit packet” containing self-help materials including: 1 week’s supply of NRT; a pictorial instruction leaflet on how to use nicotine patches; a list of resources including agencies and organizations that provide smoking cessation services; and a sample quit plan. Control group participants were provided with the self-help quit packets only. All participants were contacted between 4 and 6 months after the initial enrollment (about 3–5 months after the end of the intervention depending on the arm) to complete a follow up survey, including carbon monoxide breath testing at the community venues. All participants received cash incentives for each stage (i.e., taking the baseline community survey, attending cessation classes and participating in follow-up survey).

### Data Collection and Measures

Baseline data was collected through the community surveys using Qualtrics, a computerized web-based survey [17]. The community survey questionnaires captured information on demographics, physical and behavioral health, smoking history, barriers to quitting, readiness to quit according to the stages of change behavior model, and other variables. Participants completed check-in forms during the weekly classes and an exit form at the end of their program to document smoking status and adherence to their quit plan, and to provide information on motivators and barriers to quitting and aids for success. Three months after the intervention, follow-up questionnaires captured information on participants’ smoking status and barriers to quitting. A carbon monoxide breath monitor was used to verify the smoking status of participants at every stage [18].

At baseline, we administered the Fagerström Nicotine Dependency Test to determine the intensity of participants’ physical addiction to nicotine [19]. Scores on the Fagerström Test have a potential range from 0 (low) to 10 (high dependence), with a higher score indicating higher addiction. Smoking status was ascertained from 3 months after completion of the cessation program. The primary outcome of interest for this study was smoking status at follow-up. Participants were asked whether they currently smoke and if they had been abstinent in the past week, 1 month or 3 months. Participants were categorized as “quit” or “did not quit” based on self-reported smoking abstinence for at least 1 week, which was also validated by expired-air CO levels. A level of ≤7 ppm, commonly considered as “quit” [18, 20] showed high validity of self-reported quit data. Sociodemographic indicators included race (African American/Black, White, Native American or Alaska Native, Native Hawaiian or Other Pacific Islander), ethnicity (Hispanic or Latino), age (as a continuous variable and categorized as above or below the median age), sex (male or female), employment status (full time, part time, or unemployed), marital status (single, married or other), and educational attainment (some high school, high school graduate, some college, trade school graduate, college graduate, or more than college).

### Data Analysis

Data were exported from Qualtrics into Stata 14.0 [21] for data management and statistical analysis. Descriptive univariate analysis was conducted to review the distribution of each variable overall and in bivariate analysis by study arm. We summarized the demographic and baseline information by study arm. For bivariate analysis, we used chi-square test to compare quit rates across groups. We used this test to compare study arms by sex, race, education, marital status and employment status. One way ANOVA test was used to compare the means of continuous variables (age and Fagerström score) across groups. A multivariable logistic regression model was fitted to compare the odds of quitting at 12 weeks across groups. Odds ratios (OR) and 95% confidence intervals were reported before and after adjusting for co-variates (study arm, sex, age, race, employment, marital status, education, Fagerström). *p*-values of less than 0.05 were considered statistically significant. Participant attrition was addressed by conducting analyses using two scenarios. The primary analysis consisted of only participants who completed follow-up, while in the secondary analysis which was the more conservative approach, all enrolled participants were included and participants without follow-up data were categorized as those who did not quit.

### Ethical Considerations

This intervention involved human subjects who were recruited and followed up for a period of time. The proposal for this intervention was approved by Morgan State University’s Institutional Review Board (IRB) and the CEASE Community Action Board. Each participant signed an informed consent prior to being enrolled in the study.

## Results

**FIGURE 1:**
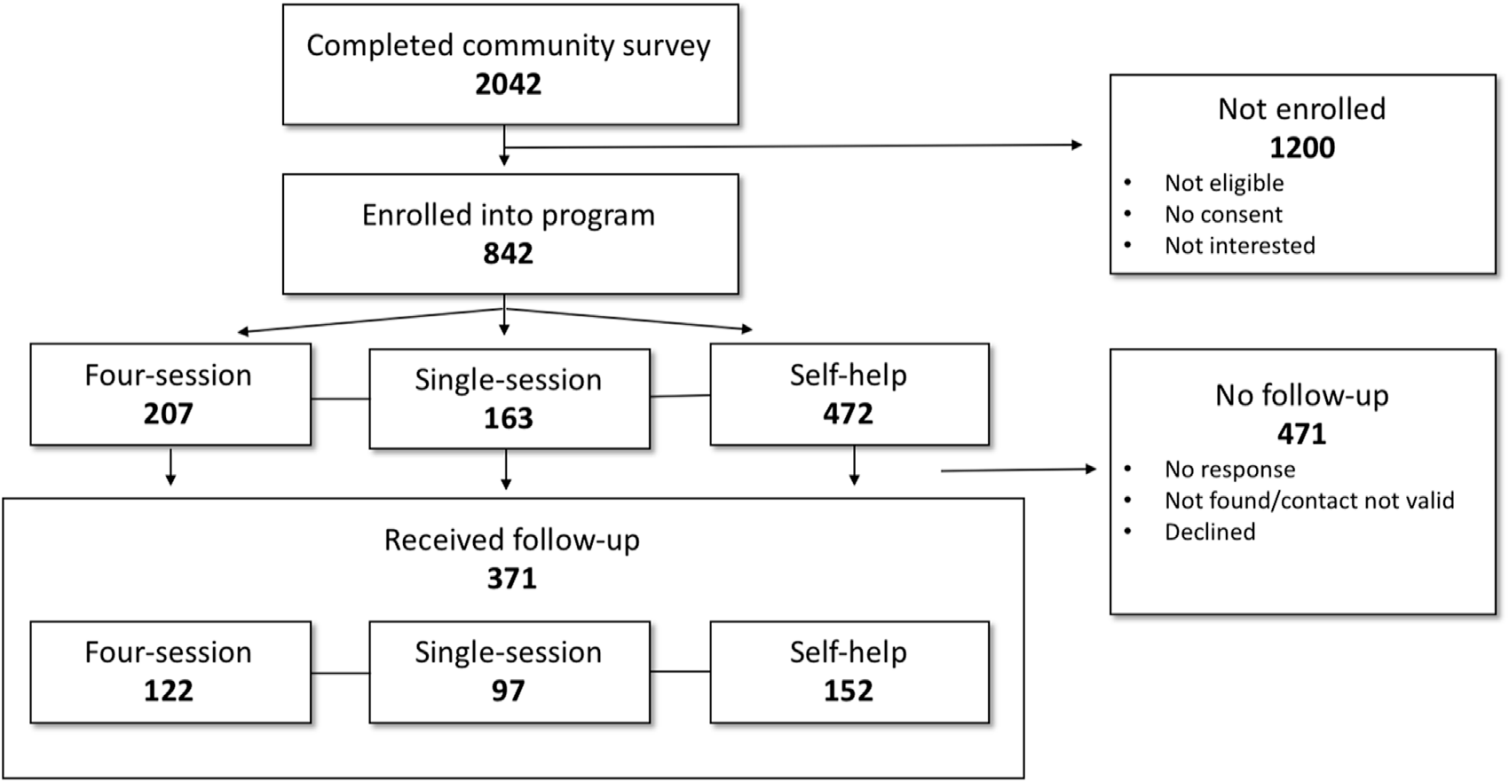
Flow chart showing number of participants at each stage of the Communities Engaged and Advocating for a Smoke-Free Environment-4 (Baltimore, United States. 2015–2017).

Of 2042 community surveys done, 842 eligible participants (41.2%) enrolled into the program. Of the enrolled participants, 472 (56.1%) received self-help smoking cessation, 163 (34.5%) attended single-session groups, and 207 (24.6%) participants attended the four-session groups (Figure 1). The number of participants who completed follow-up surveys from the four-session, single-session and self-help groups were 122 (58.9%), 97 (59.5%) and 152 (32.2%), respectively (Figure 1). The 371 participants with follow-up data comprised the primary analytic sample while outcomes for all participants are presented as secondary analyses.

### Sociodemographic and Baseline Characteristics

Table 1 shows the sociodemographic and baseline characteristics of participants who completed follow-up (*n* = 371) compared with participants who did not complete follow-up (*n* = 471).

**TABLE 1:** Sociodemographic and baseline characteristics of the Communities Engaged and Advocating for a Smoke-Free Environment −4 participants by intervention arm; comparing those who did not receive follow-up with those who received follow-up (Baltimore, United States. 2015–2017).

Variables^a^	No follow-up *n* = 471 (%)	Had follow-up *n* = 371 (%)
Sex		
Female	201 (42.7)	177 (47.7)
Male	270 (57.3)	194 (52.3)
Age^b^		
Mean (SD)	48 (11.5)	52 (11.8)
<53 years	286 (61.8)	172 (47.0)
≥53 years	177 (38.2)	194 (53.0)
Race^b^		
Black	377 (80.0)	320 (86.3)
White	79 (16.8)	39 (10.5)
Other	15 (3.2)	12 (3.2)
Employment^b^		
Full-time	82 (17.4)	37 (10.0)
Part-time	51 (10.8)	45 (12.1)
Unemployed	338 (71.8)	289 (77.9)
Marital Status		
Single	353 (75.0)	268 (72.2)
Married	54 (11.5)	56 (15.1)
Other	64 (13.6)	47 (12.7)
Education		
Some high school or less	132 (28.0)	107 (28.8)
Graduated high school/GED	224 (47.6)	175 (47.2)
Some college or more	115 (24.4)	89 (24.0)
Fagerström		
Mean (SD)	4.9 (2.2)	4.8 (2.0)
<5	202 (42.9)	157 (42.3)
≥5	269 (57.1)	214 (57.7)

^a^ A chi-square test of independence was performed for categorical variables (sex, age, race, employment, marital status, education). A one-way ANOVA test was performed for continuous variables (age and Fagerström score).

^b^ Statistically significant based on alpha level of 0.05.

The baseline characteristics of participants who completed follow-up were largely comparable to the characteristics of those who did not complete follow-up. However, participants who had follow-up were older than participants who did not have follow-up; [Mean (SD); 52 (11.8) vs. 48 (11.5), respectively]. There were more black participants among those who had follow-up [320 (86.3%)] compared to those who did not have follow-up [377 (80%)]. Additionally there were more individuals who were unemployed among participants who had follow-up [289 (77.9%)] compared to those who did not have follow-up [338 (71.8%)].

### Primary Analysis

Table 2 reports bivariate (unadjusted) and multivariable (adjusted) analyses of the factors associated with smoking cessation (quitting) among participants who received a follow-up. In this analysis, no assumptions were made about the smoking status of non-responders. Smoking cessation rates were 9.0% in self-help, 10.3% in single-session and 17.8% in the four-session arms, however, this difference was not statistically significant. In the adjusted model, compared to the self-help arm, the odds of quitting in the four-session arm were higher [OR (95% CI); 2.2 (1.0–4.9)], however, this did not reach statistical significance. The odds of quitting among individuals with a higher nicotine dependence (median Fagerström score of 5 or more) was lower compared to the odds of quitting among individuals with a lower nicotine dependence (median Fagerström score of less than 5). This finding was also not statistically significant; [OR (95% CI); 0.5 (0.3–1.0)]. Similarly, sex, age, race, employment status, marital status and educational attainment were not significantly associated with the odds of quitting.

**TABLE 2:** Logistic regression of factors associated with quitting: only participants receiving follow-up carbon monoxide test (Baltimore, United States. 2015–2017).

Variables^a^	Quit (n)	Quit (%)	Unadjusted OR (95% CI)	Adjusted^b^ OR (95% CI)
Arm				
Self-Help (*n* = 122)	11	9.0	Ref	
Single Session (*n* = 97)	10	10.3	1.2 (0.5–2.9)	1.2 (0.5–3.1)
Four Session (*n* = 152)	27	17.8	2.2 (1.0–4.6)	2.2 (1.0–4.9)
Sex				
Female (*n* = 177)	23	13.0	Ref	
Male (*n* = 194)	25	12.9	1.0 (0.5–1.8)	1.1 (0.6–2.0)
Age				
<53 years (*n* = 172)	23	13.4	Ref	
≥53 years (*n* = 194)	25	12.9	1.0 (0.5–1.8)	0.8 (0.4–1.5)
Race				
African American (*n* = 320)	43	13.4	Ref	
White (*n* = 39)	4	10.3	0.7 (0.2–2.2)	0.8 (0.3–2.5)
Other (*n* = 12)	1	8.3	0.6 (0.1–4.7)	0.5 (0.1–4.3)
Employment				
Full-time (*n* = 37)	4	10.8	Ref	
Part-time (*n* = 45)	5	11.1	1.0 (0.3–4.2)	0.9 (0.2–4.0)
Unemployed (*n* = 289)	39	13.5	1.3 (0.4–3.8)	1.0 (0.3–3.2)
Marital Status				
Single (*n* = 268)	37	13.8	Ref	
Married (*n* = 56)	6	10.7	0.7 (0.3–1.9)	0.8 (0.3–2.0)
Other (*n* = 47)	5	10.6	0.7 (0.3–2.0)	0.6 (0.2–1.8)
Education				
Some high school or less (*n* = 107)	16	15.0	Ref	
Graduated high school/GED (*n* = 175)	19	10.9	0.7 (0.3–1.4)	0.8 (0.4–1.6)
Some college or more (*n* = 89)	13	14.6	1.0 (0.4–2.1)	1.1 (0.5–2.7)
Fagerström				
<5 (*n* = 157)	26	16.6	Ref	
≥5 (*n* = 214)	22	10.3	0.6 (0.3–1.1)	0.5 (0.3–1.0)

^a^ A chi-square test of independence was performed for categorical variables (arm, sex, race, employment, marital status and education).

^b^ Adjusted for arm, sex, age, race, employment, marital status, education, Fagerström score.

^c^ Statistically significant based on alpha level of 0.05.

### Secondary Analysis

Table 3 presents the results of bivariate (unadjusted) and multivariable (adjusted) analyses of potential predictors of quit rates for all participants who enrolled in the program regardless of whether or not they received follow-up. The results in this table are based on the assumption that all of those who did not show up for follow-up did not quit. Smoking cessation rates differed significantly among the three arms with 2.3% in the self-help arm, 6.1% in the single-session arm and 13.0% in the four-session arm (*p* < 0.05). In the unadjusted model, participants in the single-session arm had almost a three times higher odds of quitting compared to those in the self-help arm [OR (95% CI); 2.7 (1.1–6.7)], while participants in four-session arm had a six times higher odds of quitting compared to the self-help arm [OR (95% CI); 6.3 (3.1–12.9)]. When adjusted for sex, age, race, employment, marital status, education and Fagerström score, this association remained significant [OR (95% CI); 3.0 (1.2–7.5) and 6.5 (3.0–13.9), respectively].

**TABLE 3:** Logistic regression of factors associated with quitting: all participants (Baltimore, United States. 2015–2017).

Variables^a^	Quit (n)	Quit (%)	Unadjusted OR (95% CI)	Adjusted^b^ OR (95% CI)
Arm				
Self-Help (*n* = 472)	11	2.3	Ref	
Single Session (*n* = 163)	10	6.1	2.7 (1.1–6.7)^c^	3.0 (1.2–7.5)^c^
Four Session (*n* = 207)	27	13.0	6.3 (3.1–12.9)^c^	6.5 (3.0–13.9)^c^
Sex				
Female (*n* = 378)	23	6.1	Ref	
Male (*n* = 464)	25	5.4	0.9 (0.5–1.6)	1.0 (0.5–1.7)
Age				
<53 years (*n* = 458)	23	5.0	Ref	
≥53 years (*n* = 371)	25	6.7	1.4 (0.8–2.4)	1.0 (0.5–1.8)
Race				
African American (*n* = 697)	43	6.2	Ref	
White (*n* = 118)	4	3.4	0.5 (0.2–1.5)	0.6 (0.2–1.8)
Other (*n* = 27)	1	3.7	0.6 (0.1–4.4)	0.7 (0.1–5.9)
Employment				
Full-time (*n* = 119)	4	3.4	Ref	
Part-time (*n* = 96)	5	5.2	1.6 (0.4–6.1)	1.1 (0.3–4.9)
Unemployed (*n* = 627)	39	6.2	1.9 (0.7–5.4)	1.1 (0.3–3.4)
Marital Status				
Single (*n* = 621)	37	6.0	Ref	
Married (*n* = 110)	6	5.6	0.9 (0.4–2.2)	0.9 (0.4–2.2)
Other (*n* = 111)	5	4.5	0.7 (0.3–1.9)	0.7 (0.2–1.8)
Education				
Some high school or less (*n* = 239)	16	6.7	Ref	
Graduated high school/GED (*n* = 399)	19	4.8	0.7 (0.4–1.4)	0.7 (0.3–1.5)
Some college or more (*n* = 204)	13	6.4	0.9 (0.4–2.0)	1.1 (0.5–2.4)
Fagerström				
<5 (*n* = 359)	26	7.2	Ref	
≥5 (*n* = 483)	22	4.6	0.6 (0.3–1.1)	0.5 (0.3–1.0)

^a^ A chi-square test of independence was performed for categorical variables (arm, sex, race, education, marital status and employment).

^b^ Adjusted for session, sex, age, race, employment, marital status, education, Fagerström score.

^c^ Statistically significant based on alpha level of 0.05.

The odds of quitting for participants with a lower nicotine dependence (median Fagerström score of less than 5) at baseline was twice the odds of quitting in participants with a higher nicotine dependence (median Fagerström score of 5 and above). This association however, was not statistically significant in the adjusted model [OR (95% CI); 0.5 (0.3–1.0)]. Sex, age, race, employment, marital status and educational attainment were not found to be significantly associated with the odds of smoking cessation.

## Discussion

Implemented in a low-income minority-majority community in Baltimore, this study had two aims. First, to test the effectiveness of the CEASE–4 intervention, and second, to explore the correlates of the response to the intervention. Despite attrition, which remained high regardless of the number of sessions, participants who remained in the study showed some promising effects, which can be attributed to the theory-based curriculum, tailoring the program to the needs of underserved populations, and delivery and design based on CBPR principles. According to our primary analysis, the observed differences in smoking cessation rates in the three study arms were not statistically significant. The results of our secondary analysis, however, showed that cessation rates were highest (13.0%) in the four-session arm, lowest (2.3%) in the self-help arm, and in between (6.1%) in the single-session arm. These smoking cessation rates were statistically different across groups; therefore our results suggest that while a CBPR theory-based smoking cessation program is effective for underserved populations, a curriculum that builds on motivation and increasing self-efficacy to quit smoking, is superior to merely educating participants. We also found that a four-session curriculum might be superior to a single session program. Our findings suggest that participants with more severe tobacco addiction possibly had greater difficulty quitting than did participants with less severe addiction. Thus tobacco addiction severity, measured using the Fagerström test, could be predictive of smoking cessation in similar programs as also demonstrated in other studies [22, 23]. Educational attainment, age, employment, and sex, however, did not seem to predict quit rate following our behavioral intervention program.

Three main factors may explain the efficacy of our intervention: the CBPR, theory-based approach, involvement of peers for support, and the culturally tailored curriculum. The CEASE initiative has important clinical and public health implications for tackling racial/ethnic and economic disparities in tobacco burden. The collective results of CEASE advocate for the application of a CBPR approach and theory-based behavioral interventions for the needs of underserved populations of smokers. Socioeconomically underserved communities have higher rates of tobacco use, lower access to treatment, and higher treatment-related stigma than the general population, particularly their higher SES non-Latino White counterparts. Collectively, CEASE has delivered interventions for more than 800 participants using a CBPR strategy which has been instrumental for building trust and mutually beneficial relationships between academia and community for over a decade.

We aimed at reducing the CEASE intervention to one and four sessions because we presumed that the length of the intervention may negatively impact retention. To prevent this barrier, in consultation with the community, we reduced the intervention sessions to four sessions. Although we recognize that this change may reduce the efficacy of the program, our four-session program is more scalable and cost-effective and thus may be a more sustainable strategy for tobacco cessation among low-SES racial and ethnic minority smokers.

One of our motivations for implementing the four-session intervention was to increase retention, which is low in long-term tobacco cessation programs [24]. Unfortunately, attrition remains a major obstacle in the cessation programs for underserved populations. For longer interventions that use 12 sessions, more than half of the participants may not complete the program [15]. In our study also, attrition was high overall, and highest for 12-session program. High attrition remains a challenge and efforts should be made to address this undesired issue in intervention research. It is shown that number of sessions attended, regardless of the treatment modality, predicts effect of the program [25]. As attrition directly reduces the efficacy of the interventions, researchers and interventionists have shown interest in improving retention [25–29]. Subsequent efforts should be made to reduce attrition in the CEASE-4 program, and future research will be needed to test whether retention significantly improved in the four-session program compared to the original 12-session program. Attrition correlates with greater nicotine dependence, poor motivation, scheduling conflicts, health concerns, being single, using other substances, and low education [25, 26, 30]. In one study among minorities, reduced drop-out from the program was related to lower income [29].

In the adjusted logistic regression of factors associated with quitting, participants with greater nicotine addiction were less likely to successfully quit smoking but no significant associations were found between sex, age, education and employment status and smoking cessation. There are studies however, reporting associations between smoking cessation and these demographic factors [5, 31–35]. A study of retention in a smoking cessation program for Latinos in the U.S. identified unemployment as one of the predictors of program completion [36]. Another study found that a younger age predicted more no-shows in a smoking cessation program for African Americans [37]. It is therefore likely that these demographic factors could influence participant retention. The limited relationship between demographic factors in our study could be the result of controlling addiction severity.

According to our secondary analysis, cessation rates in our groups ranged between 1% and 6% and 13%, showing that a four-session program may be superior to a single session program. The quit rates achieved in this intervention, although not very high, are higher than the success rates reported in some other clinical trials that have been delivered to underserved populations [37, 38].

This study had multiple limitations. First was the quasi-experimental design. More research is needed using randomized clinical trial design, given that such a design reduces selection bias and generates the highest level of evidence [39]. As previously mentioned, although we built our intervention on motivation and self-efficacy, and despite the fact that our theoretical framework relied on enhanced motivation and self-efficacy to quit smoking, we did not measure our theory-based constructs. Thus, there is a need to further research whether changes in motivation, self-efficacy, or both, better help participants to quit smoking. We also do not know to what degree availability of NRT played a role in the success of the CEASE-4 program. We need more explanatory and mediational research to test what specific theoretical constructs explain the observed changes associated with our specific curriculum, and why such effects are absent in the self-help arm. For example, it is essential to know what stage of change best predicts positive effects of similar behavioral programs. In addition, as mentioned previously, high attrition increases the likelihood of selection bias. However, as the retained group (*n* = 371) and the lost to-follow-up group (*n* = 471) were comparable on most characteristics, the effect of this limitation on our results is mitigated. Also, given this attrition rate, our primary analysis might have been underpowered (the adjusted odds ratio for the four-session arm was on the borderline of statistical significance). We therefore presented an analysis of the full sample, including the 471 participants who were lost due to attrition, as a secondary analysis. This secondary analysis was a more conservative approach and it yielded significant odds ratios when comparing the four-session and single-session to the self-help arms in the adjusted model. Finally, despite intensive efforts, we did not have data beyond the end of the 12th week after the end of the intervention. There are studies that have followed participants for longer periods. Such studies show that relapse rates are high during the first year after quitting [37, 38, 40]. There is a need to study whether treatment effects differ for individual or group interventions over time [41].

A strength of our study was an overall large sample size. Another strength included the fact that the research was conducted in an underserved community where smoking prevalence is high and there is a great need for smoking cessation services. Community trials are important because clinical trials that are conducted in clinical settings often use criteria that would actually screen out most residents of the underserved communities [42]. It is therefore essential to accept the trade-off between potential threats to internal validity in favor of external validity (data that would produce results more applicable to underserved populations). This is important, considering how interventional research has rarely focused on the populations that need it the most. Another strength of this study was that smoking cessation was validated using exhaled CO breath tests. In our study, self-reported and CO measures were similar in most cases, especially after the initial measures.

Given these strengths and limitations, this study provides suggestive evidence about CBPR-based tobacco cessation services for underserved, low-income, and ethnic minority communities. The history of medical mistrust among low-income and racial and ethnic minorities, combined with high levels of stigma may be a major challenge for their enrollment into interventions that are implemented in clinical settings [43, 44]. We have previously shown that low-SES racial and ethnic minority smokers exhibit higher success in quitting in community settings compared to clinical settings, even when provided the same intervention in both settings [8, 15, 16]. Research has shown that in addition to efficacy, recruitment is a major challenge for clinical programs, and smoking cessation programs are not exceptions to this [43, 44].

To conclude, despite promising results, we still require additional research to compare successful quit rates of programs that utilize different number of sessions for their curriculum delivery. More research is needed on efficacy of theory-based smoking cessation services for underserved low-SES ethnic minority populations. More community-based research is needed using randomized clinical trial design.

## References

[1] Centers for Disease Control and Prevention [CDC]. Current Cigarette Smoking Among Adults in the United States. Centers for Disease Control and Prevention (2023). Available from: https://www.cdc.gov/tobacco/data_statistics/fact_sheets/adult_data/cig_smoking/index.htm (Accessed May 8, 2023).

[2] National Center for Chronic Disease Prevention and Health Promotion (US) Office on Smoking and Health [NCCDPHP]. The Health Consequences of Smoking—50 Years of Progress: A Report of the Surgeon General. Atlanta, US: Centers for Disease Control and Prevention (2014). 24455788

[3] Jamal A Homa DM O'Connor E Babb SD Caraballo RS Singh T Current Cigarette Smoking Among Adults — United States, 2005–2014. Morbidity Mortality Weekly Rep (2015) 64(44):1233–1240. 10.15585/mmwr.mm6444a2 26562061

[4] Barendregt JJ Bonneux L van der Maas PJ. The Health Care Costs of Smoking. N Engl J Med (1997) 337(15):1052–7. 10.1056/NEJM199710093371506 9321534

[5] Bravin JI Bunge EL Evare B Wickham RE Pérez-Stable EJ Muñoz RF. Socioeconomic Predictors of Smoking Cessation in a Worldwide Online Smoking Cessation Trial. Internet Interv (2015) 2(4):410–8. 10.1016/j.invent.2015.10.001

[6] Haas JS Linder JA Park ER Gonzalez I Rigotti NA Klinger EV Proactive Tobacco Cessation Outreach to Smokers of Low Socioeconomic Status: A Randomized Clinical Trial. JAMA Intern Med (2015) 175(2):218–26. 10.1001/jamainternmed.2014.6674 25506771 PMC4590783

[7] Everett SA Husten CG Kann L Warren CW Sharp D Crossett L. Smoking Initiation and Smoking Patterns Among US College Students. J Am Coll Health J ACH (1999) 48(2):55–60. 10.1080/07448489909595674 10500367

[8] Apata J Sheikhattari P O’Keefe AM Kamangar F Freedman N. Examining Factors Related to Smoking Cessation in Underserved Populations: Lessons Learned from the CEASE Initiative. Baltimore: Morgan State University (2018).

[9] Zimmerman EB. Researching Health Together:Engaging Patients and Stakeholders, from Topic Identifiction to Policy Change. Thousand Oaks, California: SAGE Publications, Inc (2020).

[10] Bandura A. Social Cognitive Theory in Cultural Context. Appl Psychol (2002) 51(2):269–90. 10.1111/1464-0597.00092

[11] Bandura A. Health Promotion by Social Cognitive Means. Health Educ Behav (2004) 31(2):143–64. 10.1177/1090198104263660 15090118

[12] Bandura A. Social Cognitive Theory: An Agentic Perspective. Annu Rev Psychol (2001) 52(1):1–26. 10.1146/annurev.psych.52.1.1 11148297

[13] Prochaska JO Velicer WF. The Transtheoretical Model of Health Behavior Change. Am J Health Promot AJHP (1997) 12(1):38–48. 10.4278/0890-1171-12.1.38 10170434

[14] DiClemente CC Prochaska JO. Toward a Comprehensive, Transtheoretical Model of Change: Stages of Change and Addictive Behaviors. In: WR Miller N Heather, editors. Treating Addictive Behaviors. New York: Plenum Press (1998). p. 3–24. 10.1007/978-1-4899-1934-2_1

[15] Wagner FA Sheikhattari P Buccheri J Gunning M Bleich L Shutzman C. A Community-Based Participatory Research on Smoking Cessation Intervention for Urban Communities. J Health Care Poor Underserved (2016) 27(1):35–50. 10.1353/hpu.2016.0017 27763459 PMC6035872

[16] Sheikhattari P Apata J Kamangar F Schutzman C O'Keefe A Buccheri J Examining Smoking Cessation in a Community-Based versus Clinic-Based Intervention Using Community-Based Participatory Research. J Community Health (2016) 41:1146–52. 10.1007/s10900-016-0264-9 27688221 PMC5083217

[17] Qualtrics. The Leading Research and Experience Software (2016). Available from: https://www.qualtrics.com/ (Accessed October 17, 2017).

[18] Ryter SW Choi AMK. Carbon Monoxide in Exhaled Breath Testing and Therapeutics. J Breath Res (2013) 7(1):017111. 10.1088/1752-7155/7/1/017111 23446063 PMC3651886

[19] Heatherton TF Kozlowski LT Frecker RC Fagerström KO. The Fagerström Test for Nicotine Dependence: A revision of the Fagerström Tolerance Questionnaire. Br J Addict (1991) 86(9):1119–27. 10.1111/j.1360-0443.1991.tb01879.x 1932883

[20] Deveci SE Deveci F Açik Y Ozan AT. The Measurement of Exhaled Carbon Monoxide in Healthy Smokers and Non-smokers. Respir Med (2004) 98(6):551–6. 10.1016/j.rmed.2003.11.018 15191041

[21] StataCorp. Stata Statistical Software:Release 14. College Station, TX: StataCorp LP (2015).

[22] Diemert LM Bondy SJ Brown KS Manske S. Young Adult Smoking Cessation: Predictors of Quit Attempts and Abstinence. Am J Public Health (2013) 103(3):449–53. 10.2105/AJPH.2012.300878 23327264 PMC3673495

[23] Sargent JD Mott LA Stevens M. Predictors of Smoking Cessation in Adolescents. Arch Pediatr Adolesc Med (1998) 152(4):388–93. 10.1001/archpedi.152.4.388 9559717

[24] Bricca A Swithenbank Z Scott N Treweek S Johnston M Black N Predictors of Recruitment and Retention in Randomized Controlled Trials of Behavioural Smoking Cessation Interventions: a Systematic Review and Meta-Regression Analysis. Addict Abingdon Engl (2022) 117(2):299–311. 10.1111/add.15614 34159677

[25] Estreet A Apata J Kamangar F Schutzman C Buccheri J O'Keefe AM Improving Participants’ Retention in a Smoking Cessation Intervention Using a Community-Based Participatory Research Approach. Int J Prev Med (2017) 8:106. 10.4103/ijpvm.IJPVM_303_17 29416835 PMC5760842

[26] Curtin L Brown RA Sales SD. Determinants of Attrition from Cessation Treatment in Smokers with a History of Major Depressive Disorder. Psychol Addict Behav (2000) 14(2):134–42. 10.1037/0893-164X.14.2.134 10860112 PMC2866073

[27] Mills DE Schaefer KR Beans JA Todd MR Robinson RF Thummel KE Retention in a 6-Month Smoking Cessation Study Among Alaska Native and American Indian People. Am Indian Alsk Native Ment Health Res J Natl Cent (2022) 29(3):71–89. 10.5820/aian.2903.2022.71 PMC9552566 36178748

[28] Yancey AK Ortega AN Kumanyika SK. Effective Recruitment and Retention of Minority Research Participants. Annu Rev Public Health (2006) 27(1):1–28. 10.1146/annurev.publhealth.27.021405.102113 16533107

[29] Nevid JS Rart J Moulton JL III. Factors Predicting Participant Attrition in a Community-Based, Culturally Specific Smoking-Cessation Program for Hispanic Smokers. Health Psychol (1996) 15(3):226–9. 10.1037/0278-6133.15.3.226 8698037

[30] El-Khorazaty MN Johnson AA Kiely M El-Mohandes AAE Subramanian S Laryea HA Recruitment and Retention of Low-Income Minority Women in a Behavioral Intervention to Reduce Smoking, Depression, and Intimate Partner Violence during Pregnancy. BMC Public Health (2007) 7:233. 10.1186/1471-2458-7-233 17822526 PMC2020481

[31] Fidler J Ferguson SG Brown J Stapleton J West R. How Does Rate of Smoking Cessation Vary by Age, Gender and Social Grade? Findings from a Population Survey in England. Addiction (2013) 108(9):1680–5. 10.1111/add.12241 23668684

[32] Jarvis MJ Cohen JE Delnevo CD Giovino GA. Dispelling Myths about Gender Differences in Smoking Cessation: Population Data from the USA, Canada and Britain. Tob Control (2013) 22(5):356–60. 10.1136/tobaccocontrol-2011-050279 22649182

[33] Klumbiene J Sakyte E Petkeviciene J Prattala R Kunst AE. The Effect of Tobacco Control Policy on Smoking Cessation in Relation to Gender, Age and Education in Lithuania, 1994–2010. BMC Public Health (2015) 15:181. 10.1186/s12889-015-1525-8 25886060 PMC4349467

[34] Stewart DW Adams CE Cano MA Correa-Fernández V Li Y Waters AJ Associations between Health Literacy and Established Predictors of Smoking Cessation. Am J Public Health (2013) 103(7):e43–9. 10.2105/AJPH.2012.301062 PMC3682601 23678912

[35] Walker JF Loprinzi PD. Longitudinal Examination of Predictors of Smoking Cessation in a National Sample of U.S. Adolescent and Young Adult Smokers. Nicotine Tob Res (2014) 16(6):820–7. 10.1093/ntr/ntu005 24520129

[36] Lee CS Hayes RB McQuaid EL Borrelli B. Predictors of Retention in Smoking Cessation Treatment Among Latino Smokers in the Northeast United States. Health Educ Res (2010) 25:687–97. 10.1093/her/cyq010 20237106

[37] Ahluwalia JS Harris K Catley D Okuyemi KS Mayo MS. Sustained-release Bupropion for Smoking Cessation in African Americans: A Randomized Controlled Trial. JAMA (2002) 288(4):468–74. 10.1001/jama.288.4.468 12132977

[38] Lipkus IM Lyna PR Rimer BK. Using Tailored Interventions to Enhance Smoking Cessation Among African-Americans at a Community Health center. Nicotine Tob Res (1999) 1(1):77–85. 10.1080/14622299050011181 11072391

[39] Hariton E Locascio JJ. Randomised Controlled Trials - the Gold Standard for Effectiveness Research: Study Design: Randomised Controlled Trials. BJOG Int J Obstet Gynaecol (2018) 125(13):1716. 10.1111/1471-0528.15199 PMC6235704 29916205

[40] Prado GF Lombardi EMS Bussacos MA Arrabal-Fernandes FL Terra-Filho M Santos UP. A Real-Life Study of the Effectiveness of Different Pharmacological Approaches to the Treatment of Smoking Cessation: Re-discussing the Predictors of success. Clinics (2011) 66(1):65–71. 10.1590/S1807-59322011000100012 PMC3044573 21437438

[41] Kottke TE Battista RN DeFriese GH Brekke ML. Attributes of Successful Smoking Cessation Interventions in Medical Practice: A Meta-Analysis of 39 Controlled Trials. JAMA (1988) 259(19):2883–9. 10.1001/jama.259.19.2883 3367456

[42] Rothwell PM. External Validity of Randomised Controlled Trials: “To Whom Do the Results of This Trial Apply?”. The Lancet (2005) 365(9453):82–93. 10.1016/S0140-6736(04)17670-8 15639683

[43] Bonevski B Randell M Paul C Chapman K Twyman L Bryant J Reaching the Hard-To-Reach: a Systematic Review of Strategies for Improving Health and Medical Research with Socially Disadvantaged Groups. BMC Med Res Methodol (2014) 14:42. 10.1186/1471-2288-14-42 24669751 PMC3974746

[44] George S Duran N Norris K. A Systematic Review of Barriers and Facilitators to Minority Research Participation Among African Americans, Latinos, Asian Americans, and Pacific Islanders. Am J Public Health (2013) 104(2):e16–e31. 10.2105/AJPH.2013.301706 24328648 PMC3935672

